# Locked-in syndrome after stellate ganglion block

**DOI:** 10.4103/0019-5049.71027

**Published:** 2010

**Authors:** Ashok Jadon

**Affiliations:** Pain Relief Service, Tata Motors Hospital, Jamshedpur - 831 004, India

Sir,

Firstly, I appreciate the prompt management of patients, which avoided catastrophe even after the intra-arterial injection of the local anaesthetic solution. However, I have to make a few comments regarding their approach and put forth a few suggestions.

1. It is an accepted fact that the previously suggested paratracheal technique,[1] which authors’ have used, resulted in many serious complications. Therefore, fluoroscopic-guided techniques are standard practice today.
2. During the repeat injection, the authors used fluoroscopy and contrast with a safe and effective outcome. However, their suggestion that fluoroscopy should be used in difficult cases only is not appropriate.
3. We do not know in which patient intra-arterial injection may take place as the negative aspiration test is not foolproof.[2,3]
4. I feel that the morbidity which occurred due to the “locked-in syndrome” and other serious side-effects that may occur during stellate ganglion block could be avoided if contrast injection and fluoroscopy are used in every patient, irrespective of subjective assessment of easy or difficult case.

## References

[1] Carron H, Litwiller R (1975). Stellate ganglion block. Anesth Analg.

[2] Korevaar WC, Burney RG, Moore PA (1979). Convulsion during stellate ganglion block. A case report. Anesth Analg.

[3] Tóz M, Erodlu F, Dodru H, Uygur K, Yavuz L (2003). Transient locked-in syndrome resulting from stellate ganglion block in the treatment of patients with sudden hearing loss. Acta Anaesthesiol Scand.

